# Self-Reported Ethnicity and Genetic Ancestry in Relation to Oral Cancer and Pre-Cancer in Puerto Rico

**DOI:** 10.1371/journal.pone.0023950

**Published:** 2011-08-29

**Authors:** Esther Erdei, Huiping Sheng, Erika Maestas, Amanda Mackey, Kirsten A. White, Lin Li, Yan Dong, Justin Taylor, Marianne Berwick, Douglas E. Morse

**Affiliations:** 1 Molecular Epidemiology Laboratory of the Division of Epidemiology and Biostatistics, Department of Internal Medicine and the University of New Mexico Cancer Center, Cancer Epidemiology and Cancer Prevention Program at the University of New Mexico Health Sciences Center, School of Medicine, Albuquerque, New Mexico, United States of America; 2 Department of Epidemiology and Health Promotion, College of Dentistry, New York University, New York, New York, United States of America; Genentech Inc., United States of America

## Abstract

**Background:**

Hispanics are known to be an extremely diverse and genetically admixed ethnic group. The lack of methodologies to control for ethnicity and the unknown admixture in complex study populations of Hispanics has left a gap in understanding certain cancer disparity issues. Incidence rates for oral and pharyngeal cancer (OPC) in Puerto Rico are among the highest in the Western Hemisphere. We conducted an epidemiological study to examine risk and protective factors, in addition to possible genetic susceptibility components, for oral cancer and precancer in Puerto Rico.

**Methodology/Principal Findings:**

We recruited 310 Puerto Rico residents who had been diagnosed with either an incident oral squamous cell carcinoma, oral precancer, or benign oral condition. Participants completed an in-person interview and contributed buccal cells for DNA extraction. ABI Biosystem Taqman™ primer sets were used for genotyping 12 ancestry informative markers (AIMs). Ancestral group estimates were generated using maximum likelihood estimation software (LEADMIX), and additional principal component analysis was carried out to detect population substructures. We used unconditional logistic regression to assess the contribution of ancestry to the risk of being diagnosed with either an oral cancer or precancer while controlling for other potential confounders. The maximum likelihood estimates showed that study participants had a group average ancestry contribution of 69.9% European, 24.5% African, and 5.7% detectable Native American. The African and Indigenous American group estimates were significantly higher than anticipated. Neither self-identified ethnicity nor ancestry markers showed any significant associations with oral cancer/precancer risk in our study.

**Conclusions/Significance:**

The application of ancestry informative markers (AIMs), specifically designed for Hispanics, suggests no hidden population substructure is present based on our sampling and provides a viable approach for the evaluation and control of ancestry in future studies involving Hispanic populations.

## Introduction

According to 2000 United States Census data, 80.5 percent of Puerto Ricans considered themselves White, and 19.5 percent reported as Non-White; 8.0 percent claimed African origin (probably from West African ancestral groups, including Ibo and Yoruba people), and only 0.4 percent of Census respondents considered themselves descendants of the Puerto Rican Tainos [1]. The Tainos were a Native American tribe whose members populated the island before the start of the historical Hispanic influence [2]–[3].

It is documented that throughout the era of the Spanish empire, Puerto Ricans lived under a segregated social structure that was a construct of limited admixture of the three main ancestral population groups [2]–[3]. The existence of these social structures was recently examined by modern genomic testing technology among healthy Puerto Ricans [4].

Incidence rates for oral and pharyngeal cancer (OPC) in Puerto Rico are among the highest in the Western Hemisphere [5]–[9]. Further, ethno-regional differences have been reported in which OPC incidence and mortality rates are much higher among Hispanic men living in New York State than among US Hispanic males as a whole [10]. A possible link between ancestral genetic factors and the epidemiological evidence regarding OPC risk among Hispanics has not been investigated previously.

The use of ancestral informative markers allows for the identification of genetic patterns associated with population substructures and can be used to explore whether such markers are related to the risk of oral squamous cell carcinoma or its associated premalignant lesions. To examine risk and protective factors among the high incidence population of Puerto Rico we carried out our study supported by the United States National Institutes of Health. One of the main aims of the research project was to identify genetic susceptibility factors influenced by ethnographic differences in the Puerto Rican population. *The goal of this analysis was to summarize associations between ethnicity and the risk of both oral premalignant lesions and squamous cell carcinoma among participants in our epidemiological study in Puerto Rico.*

## Materials and Methods

### Ethics statement

The research project was approved by the Institutional Review Boards at the University of Puerto Rico, Medical Sciences Campus; New York University, and the University of New Mexico.

### Study participants

Three hundred and ten participants diagnosed with either a benign oral condition, oral hyperkeratosis or epithelial hyperplasia (HK/EH), oral epithelial dysplasia (OED), or oral squamous cell carcinoma [mean age: 59.13 (SD±12.75) years] were enrolled from 6 pathology laboratories in Puerto Rico (see Table 1). Participants provided written consent for being part of the research project and donated biological samples for DNA extraction. They also gave permission to review their oral tissue biopsy materials and corresponding H&E stained slides. Based on the latter, experienced, board-certified oral pathologists reviewed and validated each diagnosis.

**Table 1:** Distribution of demographic variables by diagnostic group.

Variable		Benign^a^n (%)	HK/EH^b^n (%)	OED^c^n (%)	SCCA^d^n (%)
**Age** (years)	30–49	52(33.6)	14(24.6)	4(13.8)	7(11.3)
	50–59	37(23.9)	15 (26.3)	5 (17.2)	9 (14.5)
	60–69	43 (27.7)	18 (31.6)	8 (27.6)	18 (29.0)
	≥70	23 (14.8)	10 (17.5)	12 (41.4)	28 (45.2)
**Gender**	Female	97 (62.6)	31 (54.4)	15 (51.7)	16 (25.8)
	Male	58 (37.4)	26 (45.6)	14 (48.3)	46 (74.2)
**Race**	White	100 (64.5)	34 (59.6)	21(72.4)	38 (61.3)
	Black	16 (10.3)	8 (14.0)	1(3.4)	9(14.5)
	Others (including Mestiza, Hispanic, Asian, Taino descendent etc.)	39 (25.2)	15 (26.3)	7 (24.1)	15 (24.2)
**Education**	<12 yrs	37 (23.9)	17 (29.8)	11(37.9)	34 (54.8)
	12 yrs / High School	29(18.7)	12 (21.0)	3 (10.3)	19 (30.6)
	>12 yrs	89(57.4)	28 (49.1)	15 (51.7)	9 (14.5)
**Smoking status**	Never smokers	99(63.9)	26(45.6)	12(41.4)	11(17.7)
	Ex-smokers	30(19.3)	11(19.3)	6(20.7)	19(30.6)
	Current smokers	26(16.8)	20(35.1)	11(37.9)	32 (51.6)
**Alcohol consumption**	Never	73(47.1)	31(54.4)	10(34.5)	15(24.2)
	0–6 drinks/week	44(28.4)	12(21.0)	10(34.5)	5(8.1)
	7–20 drinks/week	22(14.2)	6(10.5)	4(13.8)	10(16.1)
	>20 drinks/week	13(8.4)	8(14.0)	4(13.8)	32(51.6)
	Missing	3(1.9)	-	1(3.4)	-
**Total 303 (missing diagnosis = 7)**		**155 (48.1)**	**57 (17.7)**	**29 (9.0)**	**62 (19.3)**

a Benign designation includes cases of oral benign tissue conditions (e.g. suspicious sores and inflammatory conditions removed and pathologically examined) but diagnosed and evaluated by certified pathological review as benign, used as controls in this analysis.

b HK/EK category includes cases diagnosed with either an oral hyperkeratosis or epithelial hyperplasia.

c OED category includes cases of oral epithelial dysplasia.

d SCCA category includes cases diagnosed as having an squamous cell carcinoma of the oral cavity.

Participants completed a detailed epidemiologic questionnaire that assessed self-identified race/ethnicity (White, Black, Mestiza and other), lifestyle, nutritional factors (e.g. fruit and vegetable consumption), known risk factors (including alcohol consumption, tobacco use), and oral hygiene practices.

### Biological sample collection and genotyping

Buccal cell samples were collected during the period November 2003 through May 2008 from participants using six cytological brushes inside the mouth at selected sites and by subsequent mouthwash rinses for additional buccal cell collection. Participants swished with 10 ml of Scope mouthwash and then with 8 ml of distilled water to which we immediately added 2 ml of 70% ethanol to prevent bacterial and fungal growth during shipping. All biological samples were mailed to the University of New Mexico where genomic DNA was extracted using the Puregene DNA Buccal Cell Kit (Gentra Systems, Minneapolis, MN). All samples were processed according to the manufacturer's instructions. An average of 70–80 µg of primary source of genomic DNA was obtained and quantified using a NanoDrop spectrophotometer (Thermo Fisher Scientific Inc, Rockford, IL). Optical density was measured at 260 and 280 nanometers to assess DNA yield and quality. The samples were stored in −80°C freezers prior to genotyping.

Genotype results for 12 ancestry informative markers were generated using TaqMan7900 Real-Time PCR System (Applied Biosystems Inc., Carlsbad, CA) and quality assured primer sets of TaqMan SNP Genotyping Assays.

### Ancestry informative markers (AIMs)

Ancestry informative markers (AIMs) were selected based on previously published information for Hispanics [11]–[13]. Ancestry informative markers are single nucleotide polymorphisms distributed randomly across the human genome and are helpful in discriminating the genetic contributions of main parental ethnic groups. The selected AIMs were relevant to Puerto Rican parental populations: Africans, Europeans and Indigenous Americans (Table 2). They represent Indigenous American –European ancestry, European-African ancestry, and Native American –African ancestry differences. The allele frequency difference, called delta (δ) values between two parental groups, is based on frequencies of the homozygous wild allele in one parental population compared to the other ancestral population's same allele frequency [13]. In addition to the literature data, the presence and frequencies of the homozygous wild allele for all twelve markers were validated using NCBI website HapMap data to ensure an accurate and updated selection of markers. Table 2 shows in detail the ancestry informative markers used in this study.

**Table 2:** Ancestry Informative Markers (AIMs) used for ancestry evaluation (markers were selected based on Ziv et al., 2006).

dbDNP accession	Chromosomal Location	African	European	Indigenous American	p- value for deviation from Hardy-Weinberg equilibrium
**rs285**	8p21.3	**0.97**	0.52	0.45	0.27
**rs326946**	11q23.1	**0.61**	0.17	0.07	<0.001
**rs7041**	4q13.3	**0.93**	0.41	0.45	0.03
**rs930072**	5p13.2	**0.96**	0.1	0.45	0.87
**rs203096**	17q21.33	0.65	**0.72**	0.28	0.94
**rs2695**	9q21.31	**0.81**	**0.86**	0.22	0.003
**rs594689**	11q11	0.09	**0.47**	0.13	0.012
**rs2814778**	1q23.2	0	**0.99**	**0.99**	0.008
**rs2161**	7q22.1	0.44	0.3	**0.62**	<0.001
**rs2763**	7p22.3	0.14	0.16	**0.52**	0.009
**rs3340**	5q33.2	0.06	0.19	**0.65**	0.005
**rs7349**	10p11.22	0.04	**0.87**	**0.96**	<0.001

Wild type allele frequencies are reported for each ancestral group in the table.

### Statistical analysis

First, we determined frequencies of s*elf-reported ethnicity* among study participants. Next, we generated *genetic admixture estimates* to create admixture values using LEADMIX 1.0. After genotyping and allele frequency estimation, LEADMIX 1.0 (Likelihood Estimation of ADMIXture) software was used to calculate the contribution of the three main ancestral groups represented in our study sample. LEADMIX is a Fortran computer program estimating maximum likelihood for admixture proportions and genetic drift using population data collected on representative genetic markers. The software was created by Wang at the University of Oxford, Institute of Zoology, London, UK [14]. After registration, the software was downloaded, and the input file was created containing expected and detected allele frequencies of the applied 12 ancestral markers. Group-specific ancestry estimates were generated at the University of New Mexico Center for Advanced Computing core facility using ‘custom-designed’ supercomputer resources available for this project (id2010008).

Then, we compared disease and diagnosis group specific frequencies of each ancestral genetic marker to the expected allele frequencies of AIMs published in the literature. The comparison was made based on the expected frequency values of the wild type allele in each parental population [13]. We used Hardy-Weinberg equilibrium testing to estimate deviation from the expected frequency distributions. Two-sided p-values were used. Unconditional logistic regression was used to examine whether the genotype of each SNP was predictive of disease status. Finally, principal component analysis (PCA) was used to confirm that all of the 12 markers contributed evenly to the genetic structure of our population.

## Results

Based on the questionnaire responses, self-identified ethnicity was not different among people in the different diagnostic categories. Table 1 shows the four main disease categories and the number of participants in each category. Only one individual was detected in the OED group who was self-identified Black; other disease diagnoses did not show remarkable aggregation or significant deviation by ethnicity.

The maximum likelihood estimates calculated by LEADMIX software showed that our study participants had a group average of 69.89% European, 24.45% African, and 5.66% detectable Native American ancestry contribution.

When we individually examined the parental allele frequencies of AIMs among our study participants, the allele frequencies were significantly different in our Puerto Rican study participants compared to the parental groups of Europeans, Africans and Indigenous Americans; however, we did not detect any ancestry markers that would explain a significant portion of any of the disease diagnoses (Table 3).

**Table 3:** Frequency distribution of the selected AIMs (original allele frequencies are based on Ziv et al., 2006) among the Puerto Rican study participants (N = 303).

Ancestral Marker	dbDNP accession	Wild type and variant allele frequencies in ancestral groups based on published data	Wild type allele frequency among subjects with benign oral lesions	Wild type allele frequency among subjects with HK/EK, OED, or SCCA	p-value for difference between allelic frequencies in published data and participants in all diagnostic groups*
**Black**	rs285	0.97 vs. 0.03	0.51	0.49	<0.0001
	rs326944	0.61 vs. 0.39	0.52	0.48	0.007
	rs7041	0.93 vs. 0.07	0.45	0.55	<0.0001
	rs930072	0.96 vs. 0.04	0.55	0.45	<0.0001
**European**	rs203096	0.72 vs. 0.28	0.52	0.48	0.009
	rs2695	0.86 vs. 0.14	0.58	0.42	<0.0001
	rs594689	0.47 vs. 0.53	0.55	0.45	0.08
	rs2814778**	0.99 vs. 0.01	0.52.	0.48	<0.0001
**Indigenous American**	rs2162	0.62 vs. 0.38	0.52	0.48	0.02
	rs2763	0.52 vs. 0.48	0.50	0.50	0.86
	rs2814778**	0.99 vs. 0.01	0.52	0.48	<0.0001
	rs3340	0.65 vs. 0.35	0.48	0.52	<0.0001
	rs7349	0.96 vs. 0.04	0.39	0.61	<0.0001

*Weighted overall frequency of all diagnostic groups was used in comparison.

**This marker has equal frequency in both Europeans and Indigenous Americans.

Using principal component analysis (PCA) to detect ethnic sub-groups within our sample population, we did not identify any ancestry marker that showed a statistically significant contribution to an underlying population substructure.

Unconditional multiple logistic modeling was used to assess the contribution of the 12 ancestry markers and each of the main parental groups (White European, Black, and Indigenous Americans) to the risk of being diagnosed with either an oral cancer or precancer (relative to that of a benign oral condition) while controlling for other potential confounders, including age, gender, education, smoking, and alcohol consumption. In each instance, the estimated odds ratios were relatively weak and none achieved statistical significance (Table 4).

**Table 4:** Risk estimates of different oral disease categories (being diagnosed with oral soft tissue diseases and SCCA vs. benign conditions) by ancestry informative markers.

Ancestry Informative Markers (AIMs)/ Principal Components	Crude Odds Ratio±95% CIs – Unadjusted model for oral precancer/cancer vs. benign oral conditions	Adjusted model* Odds Ratio±95% CIs
rs2850/prin1	1.11; 0.89–1.36	1.11; 0.75–1.64
rs326944/prin2	0.88; 0.70–1.10	0.89; 0.58–1.38
rs7041/prin3	0.87; 0.69–1.10	1.15; 0.74–1.78
rs930072/prin4	0.93; 0.73–1.17	0.88; 0.54–1.43
rs203096/prin5	1.09; 0.86–1.39	0.98; 0.66–1.47
rs2695/prin6	0.92; 0.72–1.17	1.10; 0.73–1.67
rs594689/prin7	0.91; 0.71–1.16	0.94; 0.60–1.47
rs2814778*/prin8	0.89; 0.68–1.15	1.12; 0.72–1.72
rs2162/prin9	1.06; 0.81–1.40	0.98; 0.65–1.49
rs2763/prin10	1.13; 0.86–1.49	0.95; 0.60–1.51
rs3340/prin11	0.98; 0.74–1.32	0.64; 0.40–1.05
rs7349/prin12	0.98; 0.72–1.34	0.62; 0.37–1.04

*Model was adjusted by age, gender, self-reported race, education, smoking status (3 cetagories), alcohol consumption (4 categories).

Unconditional logistic regression models included genetic markers as principal components and common risk factors (age, gender, education, smoking, drinking).

## Discussion

The population in Puerto Rico is historically and anthropologically admixed and segregated at the same time thereby providing an opportunity to investigate whether an underlying, undetected population substructure could affect the risk of oral cancer or pre-cancer on the island. This analysis serves as a basis for our further genetic susceptibility research including variants in immune system genes and important candidate genes connected with metastatic potential in oral cancer.

None of the 12 genotyped ancestry markers showed population substructure among the participants; however, the frequencies were indicative of an admixed population status, a finding further confirmed by our group-specific maximum likelihood estimates. Our study enrolled cases (i.e., persons diagnosed with an oral precancer or cancer) and controls (persons diagnosed with a benign oral condition) through participating pathology laboratories on the island of Puerto Rico. Although we did not apply a population-based recruitment process, our detected maximum likelihood estimates were still very close to the known European contribution to the population (80.5% in 2000 year Census vs. 69.9%) and in keeping with the fact that people from the Iberian Peninsula began to populate Puerto Rico beginning in the early 1500s [2]. New 2010 US Census information shows even closer estimates as a decreased percentage of Puerto Ricans claimed that they were Whites (75.8%) and an increased percentage self-reported as Black or African-American (12.4% in 2010 from 8% in 2000) [15].

The group-specific frequency of African markers was significantly higher based on our maximum likelihood estimation than was expected based on published 2000 US Census data (24.5% vs. 8%; p<0.0001). Interestingly, the Native American ancestry contribution was much higher in our study population than any comparable population demographic data would indicate (5.7% vs. 0.4%; p<0.0001). These results point toward new venues in the study of chronic disease development among Puerto Ricans to include anthropological and social determinants.

### Limitations of the study

This research was implemented in the midst of changing health care regulations in the United States and Puerto Rico (i.e., introduction of HIPAA). Policy changes and associated uncertainties among healthcare practitioners, pathology laboratories, and the general public posed challenges to implement data collection and personal interviews with participants, and resulted in a smaller than anticipated sample size. In addition, during implementation of the study, we identified a deficit in the detection of oral premalignant lesions on the island [16]–[17] which resulted in a lower than expected enrollment in the number of persons diagnosed with oral precancerous lesions (HK/EH and OED).

Participation bias in small study samples is an important concern in molecular epidemiology. To address this issue, we made every effort to control for undetected, potential sub-groups that would have posed problems when diagnostic groups were analyzed. We found that the study sample represented the total admixed population well. Nevertheless, more research is needed, preferably by creating a larger, pooled Hispanic cohort study that would be specifically designed to address, in detail, the ancestral contributions to genetic susceptibility for oral cancer, pre-cancer and other chronic diseases.

In summary, we found that neither self-identified ethnicity nor ancestry markers showed any significant associations with oral cancer/precancer risk in our study.

Further, the application of ancestry informative markers (AIMs), specifically designed for Hispanics, provides a viable approach for the evaluation and control of ancestry in future studies involving Hispanic populations.
